# Duplication cyst and diverticulum of the stomach: A case report of an unusual association

**DOI:** 10.1002/ccr3.5403

**Published:** 2022-02-07

**Authors:** Mohamed Ben Amar, Abderrahmen Masmoudi, Amine Zouari, Zied Hadrich, Rafik Mzali

**Affiliations:** ^1^ 518993 Departement of surgery Habib Bourguiba Hospital Sfax Tunisia; ^2^ Faculty of Medicine of Sfax University of Sfax Sfax Tunisia

**Keywords:** diverticula, duplication cyst, laparoscopic surgery, stomach

## Abstract

Gastric duplication cysts are uncommon findings in adult patients. Accurate diagnosis of these cysts is difficult. Presenting symptoms are often non‐specific, and complications are rare. We report an uncommon case of a non‐communicating duplication cyst associated to a diverticula of the stomach in 38‐year‐old women.

## Graphical abstract

Gastric duplication cyst is a rare congenital disease; however, we should learn the keys to its diagnosis. Because of its possible malignant transformation, surgery should be considered regardless of the presence of symptoms.

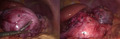

## 1 INTRODUCTION

Gastric duplication cysts are uncommon findings in adult patients. It is a rare congenital anomaly that accounts for 2–7% of all gastrointestinal duplications.^1^ Accurate diagnosis of these cysts before surgery is difficult. The presenting symptoms are often non‐specific. Complications are rare and include infection, rupture, or carcinoma arising in the cyst. Therefore, surgery should be considered especially when encountering unusual findings or suspecting malignancy transformation.^1^, ^2^ We report an unusual case of a non‐communicating duplication cyst associated with a diverticulum of the stomach in a 38‐year‐old woman.

## 2 CASE REPORT

We report the case of a 38‐year‐old Nigerian woman who presented with a two‐month history of progressively increasing epigastric pain associated with loss of appetite. Personal and family medical history was unremarkable. She was not under any medication. No smoking or alcohol drinking habits were reported. On physical examination, there was no evidence of an abdominal mass. Blood tests revealed no abnormalities. The upper endoscopy exploration showed no obvious anomaly; in particular, mucosa appeared normal and no compression was found in the antro‐pyloric region.

**FIGURE 1:**
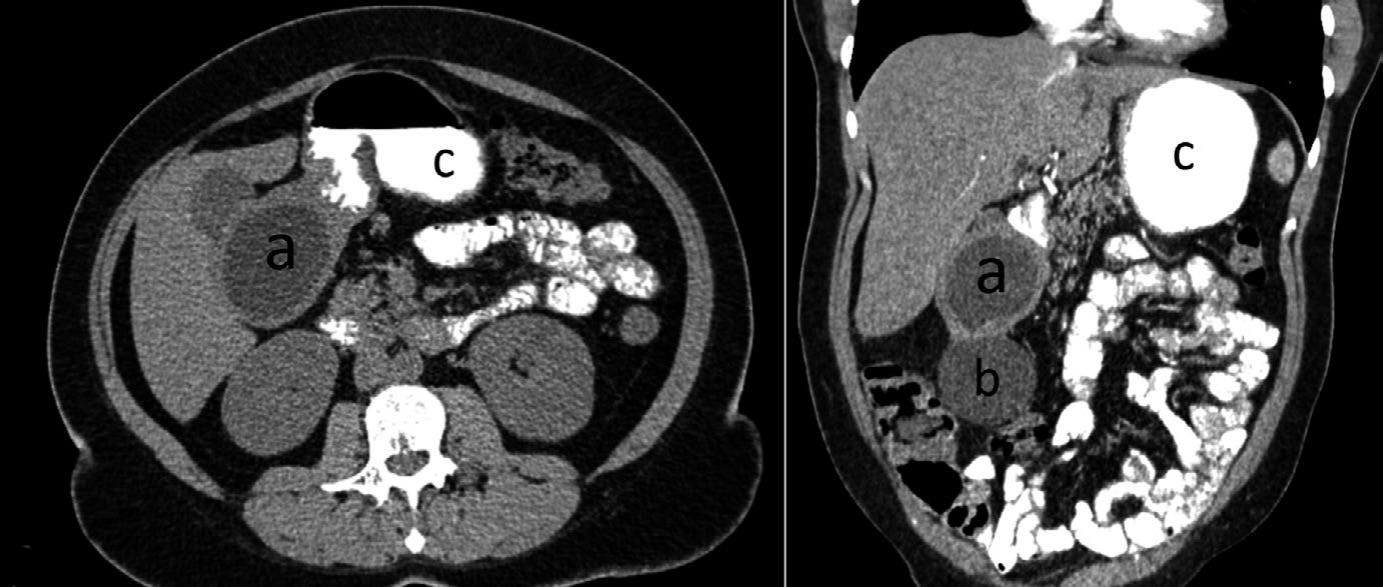
CT scan images showing the two cysts developed in the antro‐pyloric region of the stomach without communicating with its lumen

**FIGURE 2:**
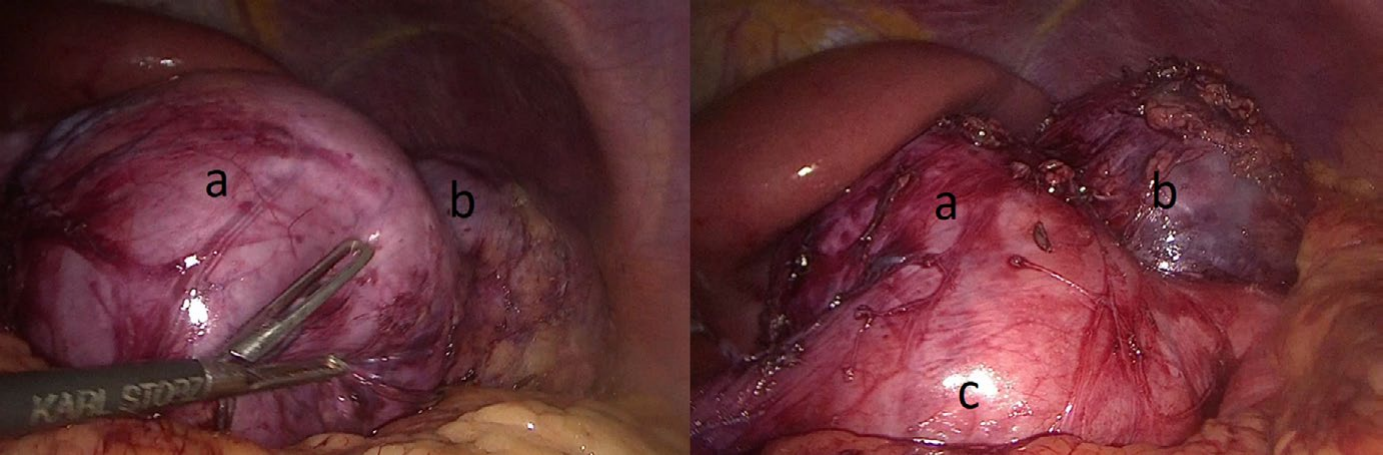
3D reconstruction, based on CT images, showing the disposition of the gastric duplication cyst

A CT scan with oral contrast material showed a 6‐cm homogeneous, non‐septated cystic mass with regular margins that could not be completely separated from the antro‐pyloric region of the stomach. The CT showed also a second cystic lesion of 7 cm contiguous to the first one. These two structures were non‐communicating with the stomach or with each other (Figure 1). A 3D reconstruction was made using the CT images (Figure 2). Initial differential diagnosis included gastric duplications cyst, pancreatic cyst, and gastrointestinal stromal tumor (GIST). A decision was made to proceed with surgery.

**FIGURE 3:**
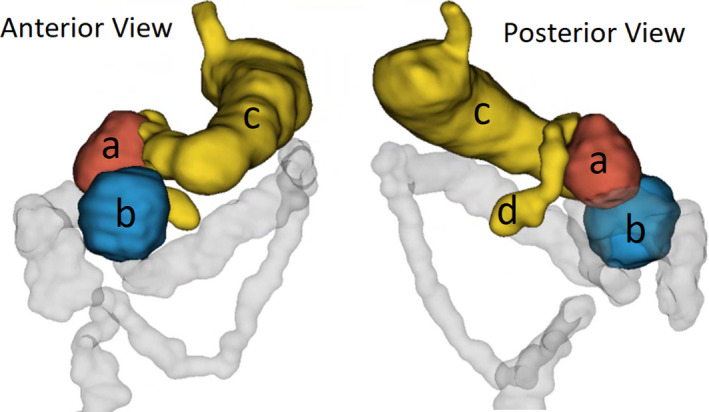
Intraoperative imaging showing the disposition of the gastric duplication

On exploratory laparoscopy, the pancreas appeared normal; however, there was a soft cystic mass measuring approximately 7 × 7 cm, which was densely adherent to the posterior wall of the stomach and arising from the antro‐pyloric region. It was separated from the gastrocolic ligament, the duodenum, and the pancreas using a Ligasure^®^ coagulating dissector. Afterward, we identified a second cystic mass of 6 × 7 cm residing in the lesser sac. It was densely adherent to the first one (Figure 3).

**FIGURE 4:**
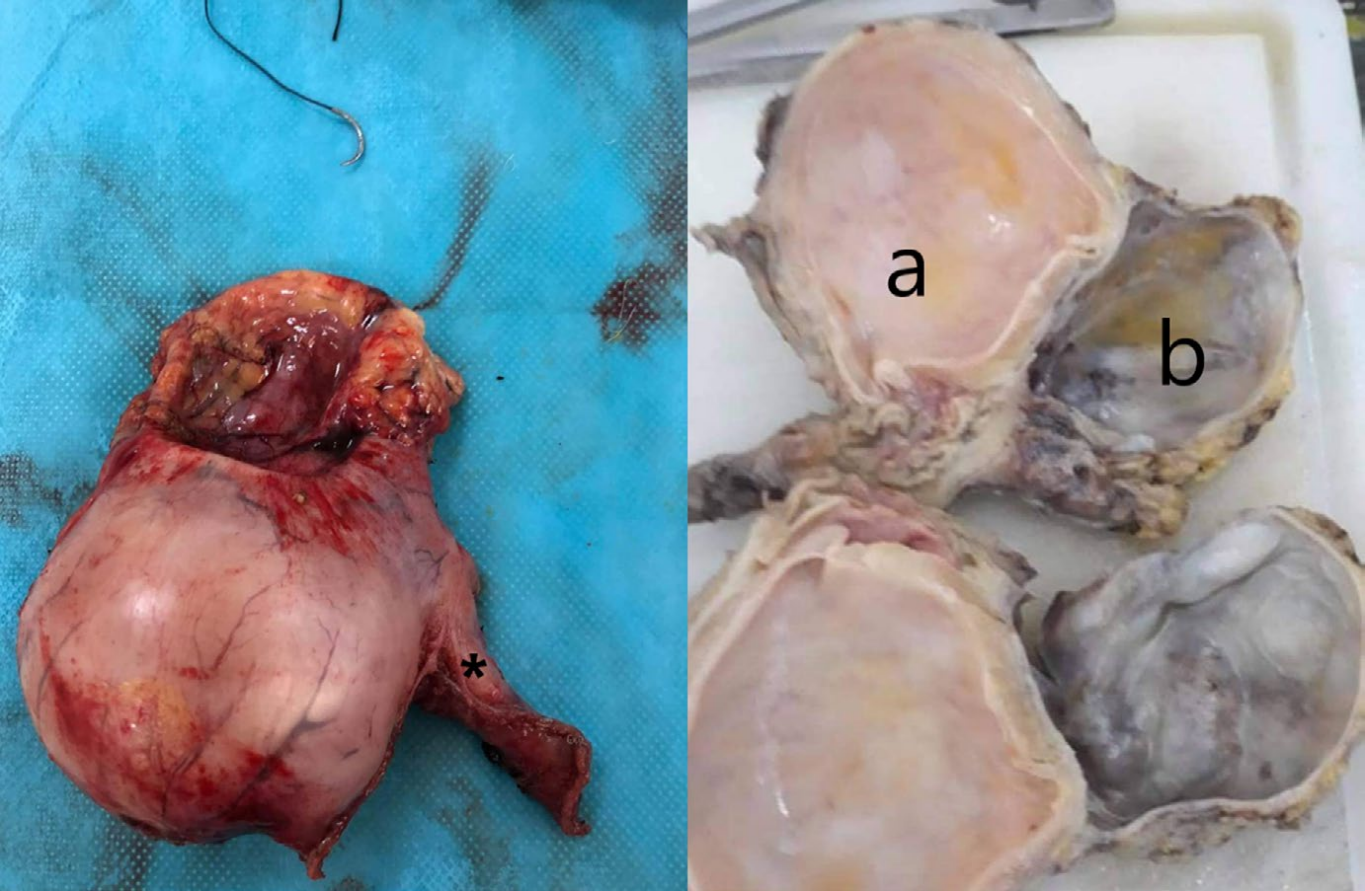
Surgical specimen showing the gastric duplication cyst

**FIGURE 5:**
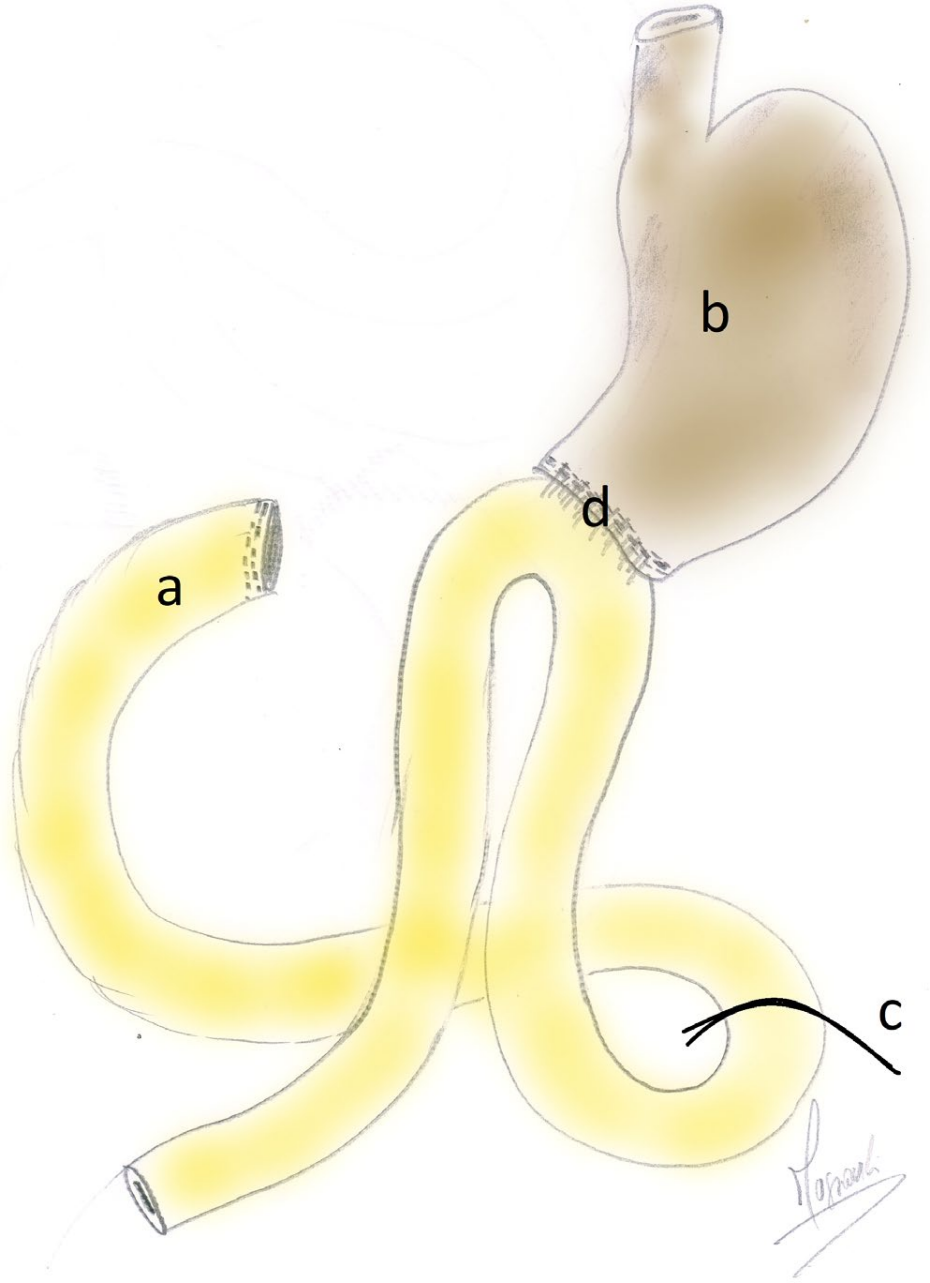
Surgical configuration after resection

After an attempt to conserve the stomach, we noticed that resection would lead to a very narrow lumen of the stomach (Figure 4). Therefore, we decided to perform a distal subtotal gastrectomy without lymph node dissection. Afterward, we performed a Billroth II reconstruction (Figure 5).

The specimen was placed in a bag and removed via a Pfannenstiel incision. The total operative time was 200 min, with minimal blood loss.

Histopathologic examination of both cavities revealed fragments with mucosal and submucosal architecture similar to the stomach (regular cubic epithelium), without dysplasia. The first cavity had a thickened muscle tissue and corresponded to a non‐communicating duplication cyst. The second was sclerotic, devoid of muscular tissue and slightly inflamed arising from the first cavity and corresponding to a diverticulum (Figure 4).

The patient started oral intake on postoperative day 1 and was discharged on postoperative day 3 without any complications.

## 3 DISCUSSION

In this report, we present a unique association of a gastric duplication cyst (GDC) and a diverticulum of the stomach in 38‐year‐old women. This association has not been reported in the literature so far.

Duplications of the alimentary tract are rare and occur in 1 in 4500 births.^3^ Usually being located in the greater curvature^4^ ^,^ GDC accounts for 2–7% of all gastrointestinal duplications.^1^ Many theories exist for the development of these lesions, including a persistent embryological diverticulum, aberrant recanalization of the alimentary tract, partial twinning, and in utero ischemic events.^5^ In most cases, diagnosis is made among pediatric populations and rarely after 12 years.^5^

By definition, gastric duplications have a well‐developed layer of smooth muscle and an epithelial lining. They are attached to the stomach sharing a common muscle wall and blood supply.^6^

Two types of duplications are described: tubular when they communicate with stomach lumen and cystic when the lumen is not contiguous with the stomach.^7^ The second type is most commonly reported. Usually, the epithelial lining is similar to the adjacent segment of the stomach but it may include, in a minority of cases, unusual cellular components such as ciliated respiratory‐type epithelium and cartilage.^8^

These cysts are usually asymptomatic and are diagnosed incidentally. However, complications can occur, including infection, bleeding, rupture, or carcinoma arising in the cyst.^1^ Symptomatic cysts have no specific symptoms. Occasionally, a palpable abdominal mass may be identified on physical examination. In the case of our patient, the only symptoms were epigastric pain and vomiting.

GDC can mimic a pancreatic pseudocyst or sometimes a malignant pancreatic cystic tumor, in particular pancreatic mucinous.^9^, ^10^, ^11^ In that case, recurrent episodes of pancreatitis have been described as first symptoms.^10^

In the past, preoperative diagnosis of gastric duplications was quite rare, but CT scan along with endoscopic ultrasound has been proven to be effective in identifying GDCs.^8^, ^10^ Classically, contrast‐enhanced CT demonstrates GDC as a thick‐walled cystic lesion with an enhancement of the inner lining.^12^ Calcifications in its wall are occasionally observed. EUS is useful in distinguishing between the intramural and extramural lesions of the stomach. Also, magnetic resonance imaging can provide a good description of the cyst content.^8^

GDC has the potential for neoplastic transformation. The production of oncofetal antigens raises the problem of a precancerous condition.^1^ Some authors favor conservative treatment because the malignant transformation of these lesions is rare, whereas others prefer complete surgical resection even in asymptomatic patients.^10^ When a malignant transformation is not suspected on the preoperative explorations, we believe that complete resection of the cyst or partial gastrectomy is the treatment of choice. However, because of the poor prognosis in the case of malignancy, we strongly recommend having an accurate diagnosis with biopsy using endoscopic ultrasonography once mural nodules can be seen in the cyst wall. When the disease is diagnosed as malignant with biopsy or after resection, subtotal or total gastrectomy with regional lymphadenectomy should be performed.^13^ In our case, the overlying gastric mucosa did not show evidence of in situ malignancy, dysplasia, or intestinal metaplasia.

On the contrary, gastric diverticula can be classified into true diverticula comprising all gastrointestinal layers and false diverticula which comprise the mucosa and the submucosa.^14^ False diverticula are usually acquired and are classified as traction or pulsion based on pathogenesis. They are generally found in the antrum and are associated with underlying inflammatory processes such as peptic ulcer disease, malignancy, pancreatitis, and gastric outlet obstruction.^14^ Its association with gastric duplication has not been reported in the literature so far. In our case, it is most likely a pulsion diverticulum caused by the underlying congenital anomaly.

## 4 CONCLUSION

GCDs are difficult to diagnose. Not only complications have been linked to this congenital anomaly, but also malignant transformation has been reported. Therefore, when unusual findings are encountered, we suggest that resection should be the first treatment option for gastric duplication cysts.

## 5 SUMMARY

This report describes a unique association of a gastric duplication cyst and a diverticulum of the stomach in 38‐year‐old women. This association has not been reported in the literature so far. Two cystic lesions were found attached to the antro‐pyloric region. We performed a distal subtotal gastrectomy with a Billroth II reconstruction. The first cavity had a thickened muscle tissue and corresponded to a non‐communicating duplication cyst of the stomach. The second corresponded to a diverticulum arising from the first cyst. Histopathologic examination showed no signs of malignancy. However, the possibility of malignancy within these cysts should always be considered.

## CONFLICTS OF INTEREST

None declared.

## AUTHOR CONTRIBUTIONS

Mohamed B.A and Abderrahmen M conceived the idea for the document and contributed to the writing and editing of the manuscript. Amine Z reviewed and edited the manuscript. Zied H reviewed and edited the manuscript. Rafik M reviewed the article before submission. All authors read and approved the final manuscript.

## ETHICAL APPROVAL

Personal data have been respected.

## CONSENT

Written informed consent was obtained from the patient to publish this report in accordance with the journal's patient consent policy.

## Data Availability

Personal data of the patient were respected. No data are available for this submission.
